# Listeria brain abscess: a therapeutically challenging rare presentation of listeriosis

**DOI:** 10.1186/s12879-024-09295-z

**Published:** 2024-05-08

**Authors:** Henrietta Bristowe, Kishan Dissanayake, Julie Chandra, Mauricio Arias

**Affiliations:** 1 Department of Infectious Sciences, King’s College Hospital NHS Foundation Trust, Denmark Hill, SE5 9RS London, England; 2 Department of Neuroradiology, King’s College Hospital NHS Foundation Trust, Denmark Hill, SE5 9RS London, England

**Keywords:** Listeria monocytogenes-brain abscess, Brain-tunnel sign, Antibiotics for brain listeriosis

## Abstract

We report a very rare case of Listeria multiple brain abscesses manifested as delirium, which represented diagnostic and therapeutic challenges overcome only by the close cooperation between Infectious Diseases and Neuroradiology, without which a satisfactory outcome would not be achieved.

An elderly man presented with confusion and drowsiness with a background of type-II diabetes mellitus. Although computed tomography of the brain only showed frontal lobe oedema, contrast magnetic resonance (MR) imaging showed numerous irregular rim-enhancing lesions containing central diffusion restriction, suggesting multiple pyogenic cerebral abscesses of unclear aetiology. Thereafter, *Listeria monocytogenes* was isolated from blood cultures, suggesting this as the causative organism. Deemed unsuitable for neurosurgical drainage, the patient received medical management with a protracted course of antibiotics. This case was extremely challenging, due to 1) the impossibility of source control, 2) the small number of effective antibiotics available to treat this condition, and 3) the inevitable antibiotic side-effects, derived from long-term exposure. A successful outcome was only possible thanks to strict close multidisciplinary follow up, requiring frequent MR imaging and a judicious antibiotic choice, including monitoring of their side-effects. Due to the rarity of this condition, there is lack of guidance on its management, hence the importance of multidisciplinary involvement with very close imaging and antibiotic monitoring.

## Introduction

*Listeria monocytogenes* is a ubiquitous Gram-positive, catalase positive, oxidase negative facultative intracellular bacterium able to replicate at low temperatures (4-8°C). Transmission mainly occurs orally through contaminated foods such as cold deli meats, unpasteurised dairy products and unwashed vegetables. Infection, referred to as listeriosis, is commonly asymptomatic or causes a mild febrile illness with gastrointestinal upset. Neurolisteriosis refers to infection of the central nervous system (CNS). It presents most commonly with meningitis but can also manifest as encephalitis, rhomboencephalitis and brain abscesses [1].

We report an unusual case of a man with delirium, which symptoms were explained by the presence of multiple cerebral abscesses. *L. monocytogenes* was subsequently isolated from blood cultures, suggesting this as the causative organism. Typical neuroimaging findings further supported this diagnosis. Management of this case was extremely challenging as there were no neurosurgical options, the patient needed complex and extended antimicrobial therapy, with associated side-effects, and required extensive imaging follow-up. The rarity of this diagnosis means treatment guidelines are lacking and drug toxicities can complicate the protracted antibiotic courses required. This case draws attention to the typical appearance of Listeria cerebral abscesses, which reflects their unique pathogenesis, as well as highlighting the paramount importance of blood cultures without which management would have been severely compromised.

## Case report

An 82-year-old man presented with a four-day history of confusion and drowsiness on a background of cognitive decline and weight loss over the preceding month. His past medical history included type-II diabetes, abdominal aortic aneurysm and monoclonal gammopathy of uncertain significance. At admission, observations and inflammatory markers were normal [C-reactive protein (CRP) 2mg/L and white blood cell count (WCC) 6x10^9^/L], and examination was unremarkable.

**Fig. 1:**
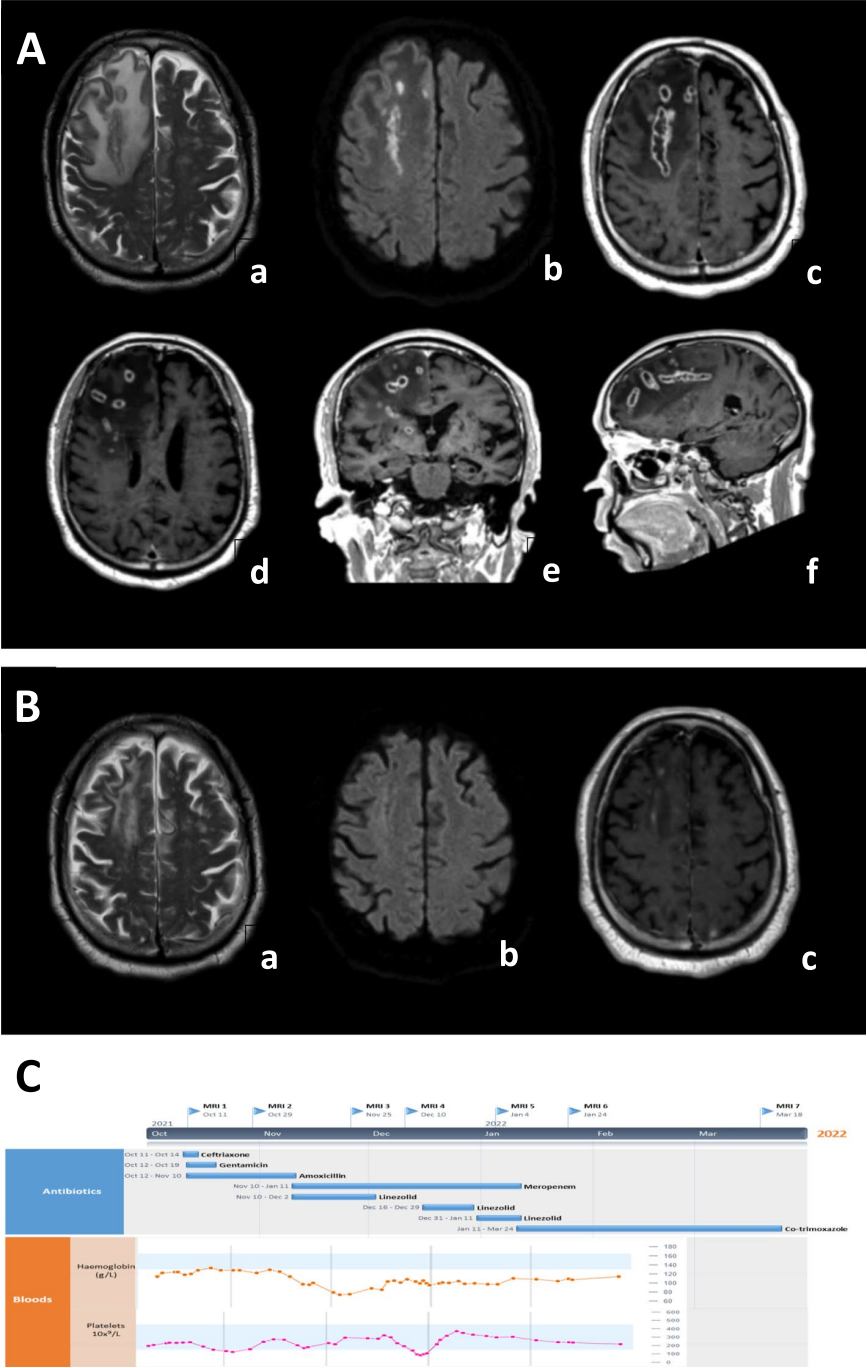
MRI scans of the brain at different stages of disease and timeline of antibiotic therapy. **A.** Initial MRI scan of the brain: axial T2-weighted image (**a**), axial diffusion-weighted image (**b**), and axial (**c** and **d**), coronal (**d**) and sagittal (**e** and** f**) post-gadolinium T1-weighted images. There are multiple linear and ovoid rim enhancing lesions in the right frontal lobe with a “tunnel sign” (**arrows**) and coalescence of smaller lesions (**arrowheads**). There is surrounding white matter T2 hyperintensity indicating vasogenic oedema and central diffusion restriction, seen as high signal, within all of the lesions suggestive of purulent content. This appearance is diagnostic for multiple bacterial abscesses, the morphology is suggestive of *Listeria sp*. **B**. Post treatment MRI brain scan at 5 months. Axial T2-weighted (**a**), axial diffusion-weighted (**b**) and post-gadolinium axial T1-weighted images (**c**) demonstrate residual mild oedema in the right frontal lobe, no diffusion restriction and minimal contrast enhancement. **C**. Antibiotic timeline in relation to timing of serial MRI brain imaging during the 125 days of hospitalisation. The patient was finally discharged at the end of February 2022 on oral co-trimoxazole. Timeline of platelets and haemoglobin in relation to antibiotics shows the transient linezolid-induced bone marrow toxicity

An unenhanced computed tomography (CT) scan of the brain revealed mild oedema in the right frontal lobe. Subsequent contrast-enhanced magnetic resonance imaging (MRI) showed numerous irregular rim-enhancing lesions containing central diffusion restriction (Fig. 1A). There were tubular components, some of which seemed to coalesce, and smaller rounded components. Moderate perilesional vasogenic oedema was noted without signs of cerebral herniation. The radiological appearances suggested pyogenic cerebral abscesses but with an unusual morphology.

On the day of the MRI scan, Gram-positive rods were isolated from blood cultures (BC). Upon admission to hospital, the patient was started on ceftriaxone 2g twice daily along with 8mg Dexamethasone twice daily. MALDI-TOF (Matrix-Assisted Laser Desorption/Ionization Time-of-Flight mass-spectrometry) identified the organism as *Listeria monocytogenes*. Typing by whole genome sequencing at the National Gastrointestinal Bacteria Reference Unit identified the isolate as serotype 4, sequence Type 1. Antimicrobial susceptibility tests showed the organism to be susceptible to amoxicillin, co-trimoxazole, and meropenem. Minimum inhibitory concentration (MIC) for linezolid and gentamicin were 4.0 and 0.19mg/L, respectively; however there are not EUCAST (European Committee for Antimicrobial Susceptibility Testing) clinical breakpoints for these two antibiotics. Chloramphenicol had a zone diameter of 14mm. At this stage, steroids were stopped and antimicrobials were switched to amoxicillin 2g four-hourly and gentamicin, the former aimed for a minimum of six weeks and the latter for seven days. Ceftriaxone lacks activity against *L. monocytogenes* and dual therapy in the early stages of treatment is recommended.

On review, the patient denied any fever or gastrointestinal symptoms. He lived with his wife who remained well, and both were vegetarian. They occasionally ate cheese but denied any recent intake of suspect food items. There was no recent animal contact or travel history. Head and neck vessel CT angiography did not reveal a source of septic emboli and transthoracic echocardiography showed neither vegetations nor valvular abnormalities. A chest, abdomen and pelvis CT scan was unremarkable. HIV and HTLV tests were negative. The scattered anatomical distribution and small size of the abscesses precluded neurosurgical drainage, so conservative management with prolonged antibiotics guided by clinical progress and neuroimaging was pursued.

Repeat BC six days into treatment confirmed clearance of the bacteraemia. Follow-up MRI after two weeks of amoxicillin therapy showed reducing size, diffusion restriction and oedema relating to the right frontal lobe abscesses but a few new enhancing lesions in the bifrontal white matter and corticospinal tracts were seen. This prompted a switch to meropenem and linezolid, which provide better CNS penetration. Unfortunately, linezolid caused bone marrow suppression requiring its suspension.

After three months of inpatient intravenous antibiotics, the decision was made to continue treatment with high dose oral co-trimoxazole (2x960mg tablets BD) with twice-weekly renal and blood count monitoring. Serial follow-up brain MRI scans showed a gradual improvement in all of the abscesses and near complete resolution after 5 months (Fig. 1B). In total, the patient completed 23 weeks of antibiotic therapy (Fig. 1C). The patient was reviewed in the outpatient clinic 6 months post completion of treatment. On this occasion, the patient was clinically very well with no evidence of neurological sequelae.

## Discussion

We describe a rare case of multiple *L. monocytogenes* cerebral abscesses in a gentleman with age and diabetes mellitus as his only predisposing factors. This case illustrates the diagnostic and therapeutic challenges arising from this central nervous system infection and supports decision-making when facing similar clinical scenarios.

Neurolisteriosis has a high mortality rate of around 25% with a specific tropism for the meninges and brainstem. It is the third leading cause of adult bacterial meningitis and the commonest aetiology of infective rhombencephalitis [2]. Importantly, genotyping of the organism demonstrated the isolate to be sequence type-1 (ST-1), which has been reported to be associated with increased neurotropism, hyperinvasiveness and enhanced proliferation capacity within macrophages when compared to environment-associated STs [3]. Complication of neurolisteriosis with cerebral abscess formation is exceedingly rare and usually associated with immunocompromise. Multiple abscesses occur in 20-25% of cases, are often located in the same cerebral hemisphere and can demonstrate an unusual tubular shape of enhancement, termed a “tunnel sign”, all features seen in this case [4]. This contrasts with more common bacterial agents such as streptococci, staphylococci and anaerobes, which typically cause rounded, ring-enhancing cerebral abscesses [5]. Listeria, as an intracellular pathogen, has the ability to infect axons thereby allowing direct linear spread of infection along the white matter fibre tracts of the brain. This explains the regional linear and coalescing pattern of abscess formation, differing from the more widespread haematogenous dissemination of infection in other bacteraemias [6]. Another intracellular pathogen, *Burkholderia pseudomallei*, the causative agent of melioidosis, creates a similar appearance on imaging by the same mechanism [7]. A final imaging mimic is sparganosis, caused by the parasite *Spirometra mansoni* in which larvae migrate through the brain extracellularly, causing tissue injury and inflammation along their way, producing a “tunnel sign” [8].

In our case, positive early BC were pivotal in identifying the pathogen informing timely antimicrobial therapy. As brain abscesses account for less than 10% of neurolisteriosis [9]with only 84 cases reported in the literature between 1968 and 2020 [10]there is limited data to inform treatment guidelines, which are based largely on the management of listeria meningitis. Linezolid, penicillin, amoxicillin, gentamicin, quinolones, meropenem, chloramphenicol and vancomycin are effective against *Listeria* species in *in vitro* studies but chloramphenicol and vancomycin have been associated with high rates of treatment failure *in vivo* [11]. The European Society of Clinical Microbiology and Infectious Diseases (ESCMID) 2016 guidelines recommend amoxicillin as first-line therapy with co-trimoxazole, moxifloxacin, meropenem and linezolid cited as alternatives. There is controversy surrounding the addition of aminoglycosides to the regimen particularly given the association with renal failure [12], as well as poor penetration into the CNS.

This case highlights many of the challenges in medical management of cerebral abscesses when drainage is not feasible. These include poor CNS penetration of amoxicillin and gentamicin in the absence of meningeal inflammation, and toxicities of certain agents when used for prolonged periods such as linezolid-associated bone marrow suppression. Most challenging here was the uncertainty of optimal treatment duration, highlighting the importance of serial imaging and close liaison with neuroradiology to guide management.

## Data Availability

No datasets were generated or analysed during the current study.
